# A Copy Paste and Semantic Segmentation-Based Approach for the Classification and Assessment of Significant Rice Diseases

**DOI:** 10.3390/plants11223174

**Published:** 2022-11-20

**Authors:** Zhiyong Li, Peng Chen, Luyu Shuai, Mantao Wang, Liang Zhang, Yuchao Wang, Jiong Mu

**Affiliations:** 1 College of Information Engineering, Sichuan Agricultural University, Ya’an 625000, China; 2 Sichuan Key Laboratory of Agricultural Information Engineering, Ya’an 625000, China; 3 College of Mechanical and Electrical Engineering, Sichuan Agricultural University, Ya’an 625000, China

**Keywords:** disease type recognition, disease level differentiation, object detection, semantic segmentation

## Abstract

The accurate segmentation of significant rice diseases and assessment of the degree of disease damage are the keys to their early diagnosis and intelligent monitoring and are the core of accurate pest control and information management. Deep learning applied to rice disease detection and segmentation can significantly improve the accuracy of disease detection and identification but requires a large number of training samples to determine the optimal parameters of the model. This study proposed a lightweight network based on copy paste and semantic segmentation for accurate disease region segmentation and severity assessment. First, a dataset for rice significant disease segmentation was selected and collated based on 3 open-source datasets, containing 450 sample images belonging to 3 categories of rice leaf bacterial blight, blast and brown spot. Then, to increase the diversity of samples, a data augmentation method, rice leaf disease copy paste (RLDCP), was proposed that expanded the collected disease samples with the concept of copy and paste. The new RSegformer model was then trained by replacing the new backbone network with the lightweight semantic segmentation network Segformer, combining the attention mechanism and changing the upsampling operator, so that the model could better balance local and global information, speed up the training process and reduce the degree of overfitting of the network. The results show that RLDCP could effectively improve the accuracy and generalisation performance of the semantic segmentation model compared with traditional data augmentation methods and could improve the MIoU of the semantic segmentation model by about 5% with a dataset only twice the size. RSegformer can achieve an 85.38% MIoU at a model size of 14.36 M. The method proposed in this paper can quickly, easily and accurately identify disease occurrence areas, their species and the degree of disease damage, providing a reference for timely and effective rice disease control.

## 1. Introduction

Rice diseases are one of the most complex, variable and insurmountable factors affecting the growth of rice, causing not only reductions in yield and quality but also food security problems. Some more severe diseases in rice production are bacterial blight, blast and brown spot [1]. Due to the ambiguity, complexity and similarity of the symptoms between different diseases, and the fact that some newbie farmers are unable to accurately diagnose and grasp the occurrence and development of rice diseases [2], quickly, efficiently and accurately detecting areas where rice diseases occur and identifying their disease types and degree of incidence to provide the necessary information for disease control has become an important issue facing rice cultivation.

Rice disease detection uses computer vision technology to detect rice disease-infested areas and their exact locations under complex natural conditions. It is a prerequisite for the accurate classification and identification of rice diseases and the assessment of disease damage levels. It is also a key to accurately locating rice disease areas and guiding plant protection equipment to target spraying. Early rice disease target detection algorithms used a sliding window strategy to select region proposals, extracted region proposal features and finally used a classifier to classify them to obtain the target area [3]. Although this method can locate disease targets without missing them, the redundant region proposal generated can be computationally intensive. It takes more time to traverse all the disease images, resulting in poor detection performance. In addition, the feature extraction of region proposal uses manual methods such as grey-scale co-occurrence matrix [4], textural descriptors [5] and local binary patterns [6], and the extracted features are more focused on the underlying features such as disease colour and shape, resulting in poor robustness of disease detection; the classifier uses support vector machines [7], Bayesian classifiers [8], unsupervised clustering [9] and other machine learning algorithms for disease recognition, with slow recognition speed and low accuracy rate.

Deep learning can automatically learn features from disease image data, which has the advantages of high learning ability, high upper-performance limit, good portability and wide coverage compared with traditional machine learning, which can avoid the limitations of manual feature engineering [10]. Datasets are the basis for building deep learning models, and the dataset’s quality determines whether the deep learning model can be trained successfully. According to the survey, several publicly available plant disease image datasets have been formed [11]. The datasets for rice disease research are fragmented, scattered and redundant, and few datasets are publicly available. Therefore, most of the existing deep learning-based plant disease diagnosis methods use data augmentation to improve the models’ recognition, detection and segmentation accuracy. The commonly used data augmentation methods are classified into traditional, supervised and unsupervised. Bhagat et al. used traditional data enhancement methods such as geometric transformation, colour transformation and fuzzy transformation to expand crop disease image data that are simple and easy to operate, but the amount of information they add is limited [12]. Therefore, the accuracy of the model is also limited. Hu et al. used SinGAN to generate many plant leaf disease images [13], but the method requires additional training overhead. The copy-paste method was proposed by snapping out instances, then dithering, flipping and pasting them onto another image, where each operation had large randomness [14]. Still, the randomness of its dithering, flipping, pasting position and number of pastes made the synthesised images challenging to understand because they did not match the actual scene.

With the rapid development of semantic segmentation models, many models have been introduced into plant disease segmentation and classification. However, it is challenging with existing models to achieve a good trade-off between accuracy and scale. Gonçalves et al. compared six pixel-level classification prediction methods and obtained relatively high accuracy with three models, FPN, UNet and DeepLabv3+ (Xception), all of which had parameter data sizes above 25 m, and SegNet, PSPNet and DeepLabv3+ (Moblienetv2) all had model parameter counts of less than 8.0 million, despite the relatively weak model generalization [15]. However, high-accuracy and lightweight models are required for accurate plant disease segmentation and easy deployment on mobile devices. Furthermore, there are many challenges with semantic segmentation models for plant foliar disease classification and segmentation studies, with the overall difficulties centred on the complexity of the context and the characteristics of the disease itself. To overcome these challenges, some researchers have improved the model architecture for plant disease segmentation and classification and produced a richer dataset [16,17,18]. Hu et al. used the UNet network model to reduce the influence of complex backgrounds on the assessment results and then used a multiconvolutional neural network model to automatically identify tea diseases in small samples [13]. Ji et al. used a two-step approach to detect grapevine black measles disease and estimate the severity to better extract disease features, first by segmenting the leaves and disease using the DeepLabv3+ semantic segmentation model based on ResNet50 and second by developing a fuzzy rule-based system for each feature to predict the degree of damage caused by the disease [19]. However, most of these models only target a single disease of a single crop in the same period and do not consider the impact of similarities between symptoms of different diseases of the same crop and changes in symptoms of the same disease of the same crop in different periods on the accuracy of the models; therefore, so the robustness of the trained models is poor, and their generalisability is weak.

Traditional plant disease severity estimation relies on manual experience. However, this method is inefficient and requires large labour and time expenditures, and the assessment results are often subjective and unreliable. In addition, more research has been carried out to automatically estimate plant disease severity by building direct models, i.e., by qualitative classification and the detection of plant disease images [20,21,22]. However, most of these models cannot achieve refined quantitative estimates, and direct models have disadvantages such as poor interpretability and weak migration performance and require retraining the model when the evaluation criteria change [23]. A novel method proposed for plant disease severity estimation is a semantic segmentation model to achieve the pixel-level classification of plant disease images and thus obtain the percentage of the lesion-to-leaf area required for plant disease severity estimation. Wang et al. proposed a two-stage model fusing DeepLabv3+ and UNet to segment cucumber leaves and disease spots based on the ratio of segmented disease spots to the leaf pixel area [24] and classified disease severity based on the percentage of segmented marks in the leaf pixel area. Chen et al. proposed a new segmentation model, BLSNet, for rice bacterial streak disease and classified severity classes based on the ratio of lesion area to total leaf area [23]. However, different plant diseases have different severity estimation criteria, and studies on the fine assessment of rice disease severity are few and limited to evaluation criteria based on area percentage, but the timely prevention and control of mid- to late-stage diseases cannot be accurately assessed with small but densely distributed disease areas.

To solve the above problems, this paper proposes a new rice leaf disease identification and segmentation model, RSegformer. The main contributions of this paper are:

(1) A publicly available dataset of common rice diseases was collected and annotated with semantic segmentation.

(2) A data enhancement method for rice disease images was proposed based on the copy-and-paste idea to generate more images that match the symptoms of rice diseases.

(3) A new rice disease segmentation model, RSegformer, was proposed, with MIoU reaching 85.38% on a parametric count of 14.36 million.

(4) An index for classifying rice leaf disease classes by combining the ratio of spots to leaf area and the number of spots is proposed to provide a valuable reference for the practical application of leaf disease severity estimation in other plants.

## 2. Materials and Methods

### 2.1. Data

#### 2.1.1. Data Acquisition

**Figure 1:**
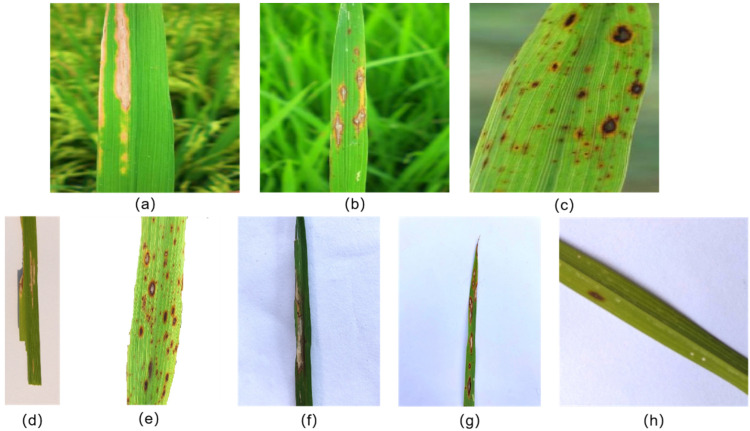
Sample images of rice diseases, (**a**) dataset 1—bacterial blight, (**b**) dataset 1—blast, (**c**) dataset 1—brown spot, (**d**) dataset 2—bacterial blight, (**e**) dataset 2—brown spot, (**f**) dataset 3—bacterial blight, (**g**) dataset 3—blast, (**h**) dataset 3—brown spot.

This dataset consists of partial data from three publicly available datasets. Dataset 1 contains 5832 images of rice leaf bacterial blight, blast, brown spot and tungro [25]. The dataset was acquired using a Nikon DSLR-D5600 camera in different rice fields in western Orissa. The paper provides images with a resolution of 300×300, from which we selected 193 original images that contain our subjects and are relatively clear. These images are unenhanced with traditional data and have large differences in image content. Dataset 2 includes 120 images of rice leaves affected by bacterial blight, brown spot and leaf blotch [2]. The dataset was taken using a NIKON D90 digital SLR camera with a white background in direct sunlight. The paper provided images with a resolution of 897×3081, from which we selected a total of 80 images containing rice bacterial leaf blight and brown spot diseases. Dataset 3 included 240 images of rice leaves affected by leaf blight, rice blast and tungro disease [26]. This dataset was taken against a white background with an image resolution of 1440×1920, and we selected 177 highly variable and clearer original images from this dataset. It is worth noting that since the least amount of data we obtained was found in the preliminary data collation session for the rice leaf disease category of rice blast, which specifically had 150 images, we determined 150 images for each disease category in order to balance the amount of data for different disease categories. Table 1 shows the amount of sample data collected for each disease category in the three datasets. Figure 1 shows examples of images for each disease type in all datasets.

#### 2.1.2. Data Annotation

**Figure 2:**
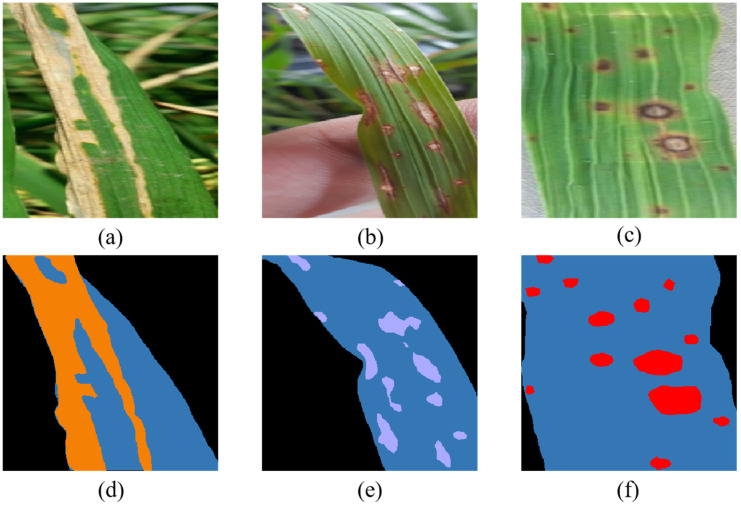
Sample rice leaf disease image and segmentation label: (**a**) bacterial blight, (**b**) blast, (**c**) brown spot, (**d**) bacterial blight label, (**e**) blast label, (**f**) brown spot label, where orange, mauve, red, blue and black represent rice bacterial blight, blast, brown spot, healthy leaves and background areas, respectively.

The dataset used for this work consisted of 450 images. Of these, 150 images were of each of the three types of diseases: rice bacterial blight, rice blast and brown spot. Considering the inconsistent resolution of different data, in order to facilitate data augmentation, all images were resized to 640×640 pixels, and 450 images were annotated using the EISeg annotation software [27], some of which are shown in Figure 2.

#### 2.1.3. Data Augmentation

To avoid model overfitting and improve model generalisation, we proposed a data enhancement method based on the idea of copy-paste called rice leaf disease copy paste (RLDCP). The RLDCP algorithm is as follows:

① Select a set of rice leaf disease images and their corresponding mask maps, noted as “org1-image” and “org1-mask”, respectively.
② A randomly selected set of images and their corresponding mask maps from the same disease dataset are noted as “org2-image” and “org2-mask”, respectively.
③ Use the edge detection operator Canny to obtain the edges of the leaves in “org1-mask” and “org2-mask” and find the minimum outer rectangle based on the edges obtained, then calculate the rotation angle of the minimum external rectangle $\alpha_{1}$ and $\alpha_{2}$ and rotate “org2-mask” and “org2-image” by $\alpha_{2}-\alpha_{1}$.
④ Key out all the lesioned pixel points according to the RGB difference of “org2-mask”, paste them into the nonbackground area on “org1-mask”, key out the pixel points on the same position of “org2-image” and “org2-mask” and paste them into “org1-image”, thus composing a new “res-image” and the corresponding “res-mask”.
⑤ Random flipping, horizontal flipping and random largescale dithering were used for the synthetic set of rice disease data “res-image” and “res-mask”.

**Figure 3:**
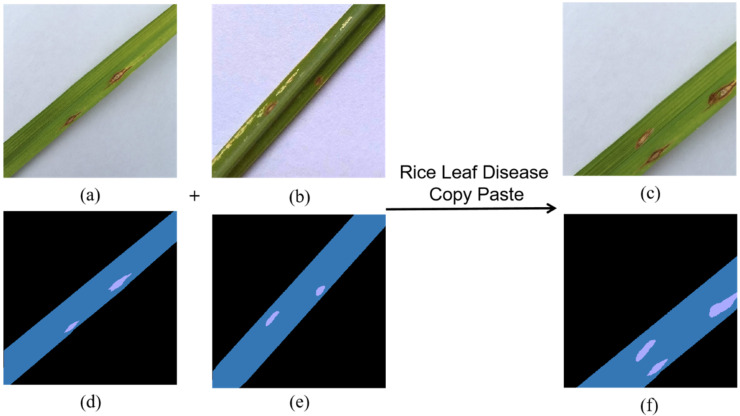
RLDCP data enhancement example: (**a**) RGB image of the pasted object, (**b**) RGB image of the copied object, (**c**) newly synthesised RGB image, (**d**) mask image of the pasted object, (**e**) mask image of the copied object, (**f**) newly synthesised mask image.

The method has both randomness and restriction in steps ② to ⑤; for instance, ② can randomly select the object to be copied. Still, the object must be the same kind of disease. In ③, you can make the distribution direction of the disease in the composite image more in line with the actual symptoms of the disease so that the leaves in the two sets of images keep the same direction by rotation but can choose to rotate to $\alpha_{2}-\alpha_{1}$ or $180^{{}^{\circ}}-\left(\alpha_{2}-\alpha_{1}\right)$. In ④, you can randomly select the range of keying, the starting position of paste and paste the number of times but must be in the nonbackground area. In ⑤, you can choose whether to flip horizontally, rotate ($0^{{}^{\circ}}\sim 180^{{}^{\circ}})$ Jitter (−1.0~2.0) or manipulate the scale of the composite image, but the new set of images generated must contain both the disease and the leaf area. Otherwise, this operation is performed again. The effect of the data after using the RLDCP data enhancement method is shown in Figure 3.

#### 2.1.4. Rice Leaf Disease Severity Label

**Figure 4:**
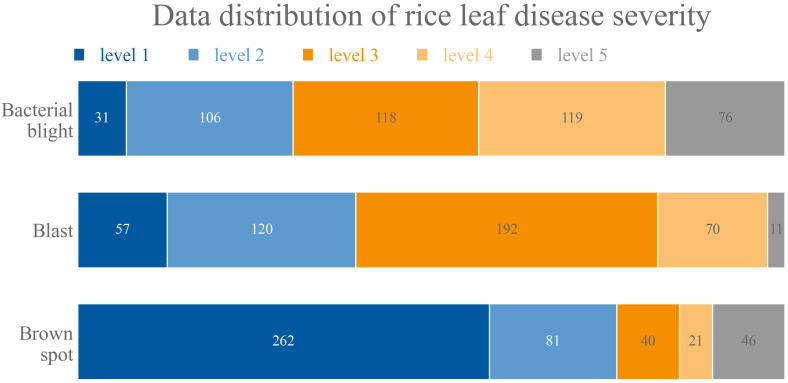
Distribution of five severity levels of three diseases in the rice leaf disease dataset.

Different criteria for measuring disease severity were designed for different disease types to solve the problem of multiple spots with a small total area covered, as in the rice brown spot in the middle and late stages of disease development. For rice bacterial blight and rice blast, the criteria are based on the percentage of the total leaf area covered by the lesion; for rice brown spot, the criteria are based on the percentage of the area covered by the lesion and the number of lesions of which the higher level is selected as the final level. In the area-based criteria, grade 0 is for healthy leaves without disease, grade 1 is for those with 0.1% to 10% lesion coverage, grade 2 is for those with 11% to 25%, grade 3 is for those with 26% to 45%, grade 4 is for those with 46% to 65% and grade 5 is for those with more than 65%. In the criteria based on the number of measurements: grade 0 is a healthy leaf without disease, grade 1 is for 1–5 spots in a single image, grade 2 for 6–10, grade 3 for 11–15, grade 4 for 16–20 and grade 5 for greater than 25. Figure 4 shows the distribution of the severity levels of the rice leaf disease dataset according to the above classification criteria.

### 2.2. Model Architecture

#### 2.2.1. Model Architecture Overview

This semantic segmentation and image classification are very much related, and semantic segmentation can be seen as an extension of image classification from the image level to the pixel level. In fact, since FCN [28], many semantic segmentation frameworks have been derived from image classification variants of ImageNet [29]. Some current semantic segmentation networks based on the convolutional neural network family adopt different networks as the feature extraction backbone, such as VGG [30], ResNet [31] and MobileNetv2 [32], or design modules and methods such as dilated convolution [33], atrous spatial pyramid pooling [34], cross-attention mechanisms [35] and point-space attention [36] to expand the perceptual field to obtain rich contextual information. However, these methods introduce many empirical modules, making the resulting framework computationally intensive and complex. With the rapid development of transformers [37] in the field of computer vision, the use of transformers as the backbone of networks to effectively expand the perceptual field to extract rich feature information through self-attentive mechanisms is one of the mainstream approaches, of which Segformer [38] is one of the typical representatives of this method applied to semantic segmentation tasks.

**Figure 5:**
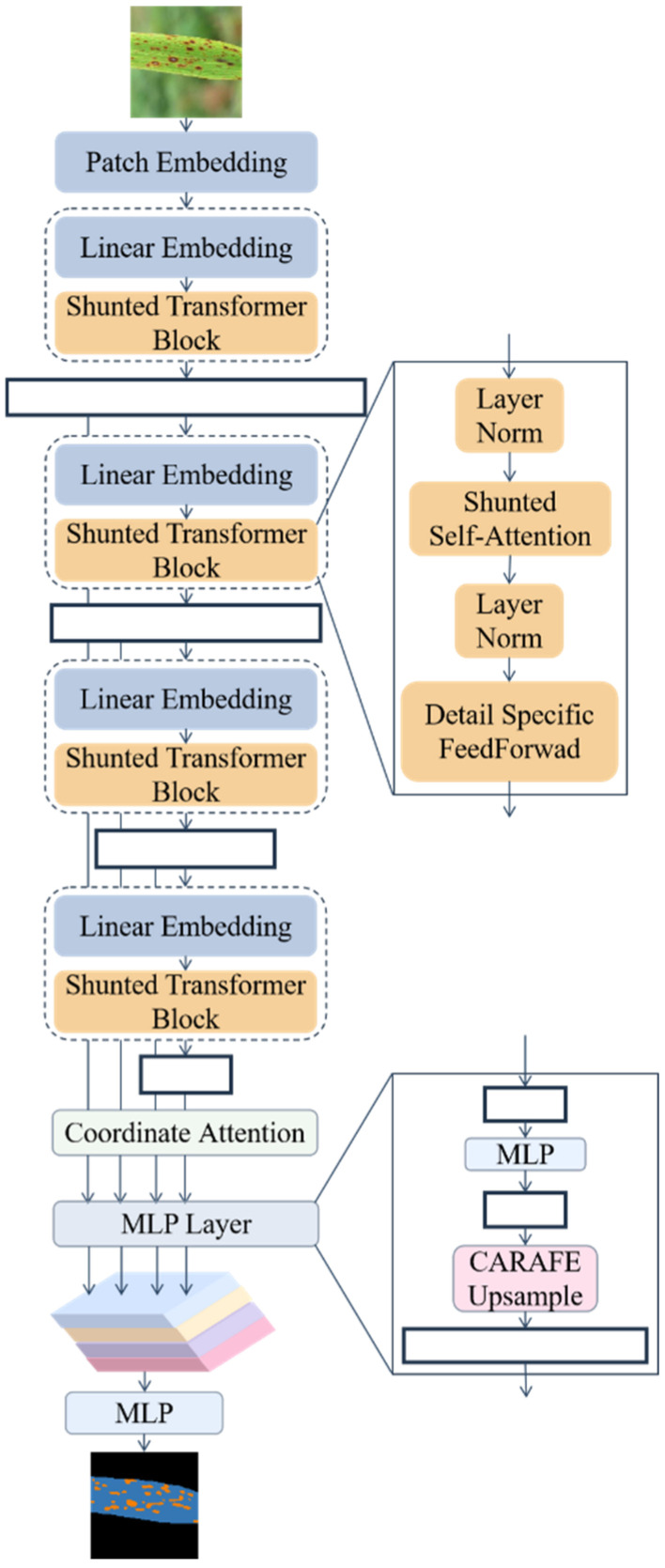
The overall architecture of the RSegformer network.

Segformer discards positional encoding, uses a novel multilevel transformer as the encoding structure to output multiscale features and uses a simple and lightweight multilayer perceptron (MLP) as the decoder to combine local and global attention to show good segmentation performance. However, the model specifies a similar field of perception for each token feature within each layer, and this constraint inevitably limits the ability of each self-attentive layer to capture multiscale features. The shunted transformer [39] proposes a novel shunted self-attentive that unifies multiscale feature extraction within a single self-attentive layer through multiscale token aggregation. In addition, in Segformer’s decoder, up-sampling using bilinear interpolation is computationally intensive, and the recovered image edges become blurred to a certain extent. The lightweight up-sampling operator Content-Aware ReAssembly of Features (CARAFE) [40] can better solve this problem. In this study, we designed RSegformer, a lightweight and efficient rice leaf disease segmentation model based on Segformer and combined shunted transformer, coordinate attention (CA) [41] and CARAFE. Figure 5 shows the overall network model architecture of RSegformer.

Similar to the architecture of Segformer, RSegformer is divided into two parts: encoding and decoding. The encoding part extracts multiscale features through four shunted transformer blocks and subsequently embeds CA attention into the encoder–decoder connection part. In contrast, the decoding part restores the feature map to the original image size via the CARAFE up-sampling operator.

#### 2.2.2. Encoding Section

Different criteria for measuring disease severity were designed for different disease types to solve the problem of multiple spots with a small total area covered, as in the rice brown spot in the middle and late stage.

(1) Shunted transformer block

The shunted transformer block consists of shunted self-attention and a detail-specific feed-forward layer.

Shunted self-attention (SSA) in the shunted transformer block: SSA divides multiple attention heads within the same layer into groups, each of which explains a specific granularity of features by aggregating a different number of tokens before calculating the self-attention matrix, thus enabling different attention heads within the same layer to simultaneously allow objects of various scales to be modelled efficiently and simultaneously on different attention heads within the same layer. The SSA calculation can be expressed as (1)–(4):

(1) $$Q_{i}=XW_{i}^{Q}\;$$

(2) $$K_{i},V_{i}=MTA\left(X,r_{i}\right)W_{i}^{K},MTA\left(X,r_{i}\right)W_{i}{}^{V}\;$$

(3) $$V_{i}=V_{i}+LE\left(V_{i}\right)\;$$

(4) $$h_{i}=Softmax\left(\frac{Q_{i}K_{i}{}^{T}}{\sqrt{d_{h}}}\right)V_{i}\;$$

In the above equations, i denotes the ith head, $W_{i}{}^{Q},W_{i}{}^{K},W_{i}{}^{V}$ is the linear projection layer parameter of the ith head, $r_{i}$ denotes the downsampling rate, MTA(⋅)  denotes the token aggregation in the ith head, LE(⋅) the locally augmented component of the value V by deep convolution of MTA(⋅), and $d_{h}$ denotes a vector dimension of query and key. The input sequence $X=R^{h\times w\times c}$ is first projected into the $Q_{i},K_{i},V_{i}$ tensor via the $W_{i}{}^{Q},W_{i}{}^{K},W_{i}{}^{V}$ linear mapping parameter, where $K_{i},V_{i}$ is downsampled to different sizes by convolutional layers of kernel and stride size $r_{i}$ and then aggregated at multiple scales by MTA(⋅). Next, $V_{i}$ is added to the locally enhanced component obtained by deep convolution using via LE(⋅). Finally, the output $h_{i}$ is obtained by performing a self-attentive calculation of $Q_{i}$ with at different scales $K_{i},V_{i}$.

Detail-specific feed-forward in the shunted transformer block: In the detail-specific feed-forward layer, to learn the cross-token information, a depth-separated convolutional branch is added to the original features before the activation layer in the two fully connected layers to enhance the connection of adjacent pixels and thus supplement the local detail information, as shown in Equations (5) and (6):

(5) $$x^{\prime }=FC\left(x;\theta_{1}\right)\;$$

(6) $$x^{\prime \prime }=FC\left(\sigma \left(x^{\prime }+DS\left(x^{\prime };\theta \right)\right);\theta_{2}\right)\;$$

where $\theta_{1}$ and $\theta_{2}$ represent the output dimensions of the first and second fully connected layers, respectively, and DS(⋅)  illustrates a detail-specific layer with parameters θ implemented by deep-separated convolution.

(2) Encoding process

Given an input image of size H×W×3, the image is first transformed into a sequence of tokens containing more valid information using the patch embedding mechanism. The length of the sequence is $\left(H\times 4^{-1}\right)\times \left(W\times 4^{-1}\right)$, and the dimensionality of each token vector is C. Patch embedding uses multiple layers of convolution, each of which includes a specific convolution, BatchNorm2d and the ReLU activation function. The first layer uses a kernel=7×7,stride=2,padding=3 convolutional layer; the second layer stacks zero or multiple kernel=3×3,stride=2,padding=1 convolutional layers depending on the required model size; and finally, a two-dimensional convolutional mapping with kernel=2×2,stride=2 generates an input sequence of length $\left(H\times 4^{-1}\right)\times \left(W\times 4^{-1}\right)$.

The token sequence is sequentially entered into four stages to obtain multiscale feature information, each containing a linear embedding and multiple shunted transformer blocks. The linear embedding uses a convolutional layer with a stride size of 2 to achieve down-sampling, while each shunted transformer block outputs a feature map of the same size. Thus, four feature maps are obtained at $F_{1},F_{2},F_{3},F_{4}$, and each stage outputs a feature map of the size of $F_{i}$ at $\left(H\times 2^{-\left(i+1\right)}\right)\times \left(W\times 2^{-\left(i+1\right)}\right)\times \left(C\times 2^{i-1}\right)$. Table 2 shows the parameter settings for the different stages.

#### 2.2.3. Decoding Section

(1) Coordinate Attention

CA consists of two parts: position attention encoding and position attention generation. In the position attention encoding stage, the input feature map of shape C×H×W  is encoded for each channel in both width and height directions to generate a feature map with a global perceptual field. In the location attention generation part, the two feature maps are stitched together. The stitched feature map goes through the convolution layer of 1×1, batch normalisation and the activation layer to obtain the feature map F of shape $C\times r^{-1}\times 1\times \left(W+H\right).$ Then the F is split into two independent tensors $F_{h}$ and $F_{w}$ along the spatial dimension, which is transformed by the convolution of 1×1 into a tensor with the same number of channels as the input feature map. The final feature map with attention weights in the width and height directions is obtained by multiplying and weighting the original feature map.

(2) Content-Aware ReAssembly of Features

CARAFE is divided into two modules, the up-sampling kernel prediction module and the feature reassembly module. Assuming an up-sampling multiplier of σ and given an input feature map of the shape H×W×C, after the up-sampling kernel is predicted, the feature reassembly module is used to complete the up-sampling to obtain an output feature map of the shape σH×σW×C.

(3) Decoding process

If the graded transformer as an encoder has a larger acceptance domain than the CNN as an encoder, the decoding part is composed of a lightweight decoder consisting of only MLP layers. The all-MLP decoder consists of four main steps, first unifying the channel dimension by passing the multilevel features $F_{i}$ obtained from the encoder shunted transformer through an MLP layer, then using the CARAFE operator to up-sample the multilevel features to $\left(H\times 4^{-1}\right)\times \left(W\times 4^{-1}\right)$, followed by fusing the connected features using MLP and finally using the MLP prediction segmentation mask. Equations (7)–(10) can express the decoding part:

(7) $$\hat{F_{i}}=Linear\left(C_{I},C\right)\left(F_{I}\right),\forall i\;$$

(8) $$\hat{F_{i}}=CARAFE\left(\frac{W}{4}\times \frac{W}{4}\right)\left(\hat{F_{i}}\right),\forall i\;$$

(9) $$F=Linear\left(4C,C\right)\left(Concat\left(\hat{F_{i}}\right)\right),\forall i\;$$

(10) $$M=Linear\left(C,N_{cls}\right)\left(F\right)\;$$

where CARAFE(⋅) is the up-sampling operation for the feature map using the CARAFE operator, $N_{cls}$ is the number of categories and M is the final prediction segmentation mask obtained.

## 3. Experimental Process

### 3.1. Realisation Details

Our model was trained using 128 GB of memory powered by a Quadro RTX5000 graphics processing unit (GPU) under the Ubuntu20.04 LTS system environment. In order to validate the effectiveness of the data augmentation method, the PSPNet [42], HRNet [43] and OCRNet [44] networks were used to train raw data, traditionally augmented data and RLDCP augmented data, respectively. To verify the validity of the models, data obtained by RLDCP augmentation were used, trained with models of similar size (DeepLabv3+ model with ResNet18 as the backbone and Segformer model with MiT-B1 as the backbone). All of the experimental models used in our comparison experiments were derived from the MMSegmentation [45] codebase. Therefore, the pretraining weights and hyperparameters for the comparison experiments inherited the default settings from MMSegmentation, with a training image size of 512×512. Furthermore, the model proposed in this paper is also based on the MMSegmentation codebase implementation. The pretraining weights used are obtained from the shunted transformer backbone trained on the ImageNet-1k dataset. For this model, we inherited the default settings of MMSegmentation and the shunted transformer: an initial learning rate of 0.00006, a “poly” learning strategy with a default factor of 1.0, and 80k iterations using the Adam-W optimiser. In addition, the batch size during training and validation was set to 2, and the results were evaluated every 500 iterations using a multiclass cross-entropy loss function to calculate the loss, as shown in Equation (11):

(11) $$Loss=-\frac{1}{K}\overset{K}{\underset{n=1}{\sum }}\left[y_{n}log\hat{y}\right]\;$$

where $y_{n}$ indicates the pixel point accurate class label, $\hat{y}$ indicates the pixel point predicted class label and K indicates the total number of classes.

### 3.2. Assessment Indicators

We used IoU and MIoU as performance evaluation metrics for semantic segmentation models. Intersection over union (IoU): This is used to calculate the proportion of meetings and mergers between the model’s predicted and actual values for a given category, as shown in Equation (12):

(12) $$IoU=\frac{T\;\cap^{\;}P}{T\;\cup^{\;}P}\;$$

where T denotes the labelled mask map and P denotes the predicted mask map.

Mean intersection over union (MIoU): Calculates the ratio of the intersection of the model’s predicted outcomes and the true values for each category to the merged set, summing and then averaging the results, as shown in Equation (13):

(13) $$MIoU=\frac{1}{k+1}\overset{k}{\underset{i=0}{\sum }}\frac{p_{ii}}{\sum_{j=0}^{k}p_{ij}+\sum_{j=0}^{k}p_{ji}-p_{ii}}\;$$

where $p_{ij}$ denotes quantities that were originally in the class but were predicted to be in the class, $p_{ji}$ denotes amounts that were originally in the class but were predicted to be in the class, $p_{ii}$ denotes true quantities, $p_{ij},p_{ji}$ is interpreted as false positive and false negative, respectively, and k denotes class numbers.

## 4. Discussion

### 4.1. Validation of Data Augmentation Methods

In this experiment, we chose three of the more popular network models, namely PSPNet, HRNet and OCRNet. To verify the effectiveness and superiority of our proposed data augmentation method and to validate the performance change when RLDCP was used a different number of times, we compared the segmentation accuracy of four datasets (the original dataset, the dataset obtained after running the traditional data augmentation method twice and the dataset obtained after running the RLDCP augmentation method once and then twice) on three classical semantic segmentation models, using MIoU as the evaluation metric. The original dataset contained 450 disease images, and with each data enhancement, the number of datasets increased by 450. Thus, a single data augmentation produced a dataset with 900 disease images and double data augmentation produced a dataset with 1350 disease images. It is worth noting that in order to obtain more valid information from the original image by traditional data augmentation methods, we chose two classical traditional data augmentation methods, namely random rotation and the addition of pretzel noise. In particular, we divided the data within each level of the three diseases in turn in a ratio of 8:2 to form the training and validation sets required for our experiments. The experimental results are shown in Table 3. We found that the MIOU values of the dataset enhanced using the RLDCP method increased on the different network models, demonstrating the effectiveness of RLDCP in the segmentation process.

Observation of Table 3 revealed that (1) traditional data enhancement methods reduced the segmentation performance of the model. We analysed the reason for this, probably because the datasets we used originated from three different environments with widely varying data distributions. The limited amount of information added by random rotation and pretzel noise amplified this imbalance by repeating memory on the data. (2) The RLDCP data enhancement method effectively improves model segmentation accuracy. Compared with the original datasets PSPNet, HRNet and OCRNet, MIoU improved by 5.47%, 6.34% and 5.04% after two RLDCP data augmentations, respectively. We analyse that this may be because the rice leaf disease copy paste method synthesises reasonable rice leaf disease images by restricted copy-paste, which effectively expands the sample data volume, reduces the impact of data distribution differences and improves the generalisability of the model. (3) Training with the data augmented with one RLDCP data augmentation, MIOU increased significantly for all three models. When we added another RLDCP data augmentation, MIOU also increased for all three models. We believe that as the amount of data increases, MIoU will tend to saturate. Although the traditional data enhancement method also increased the amount of data, it did not increase the MIOU, so our proposed RLDCP method is effective.

### 4.2. Model Comparison Experiments

**Figure 6:**
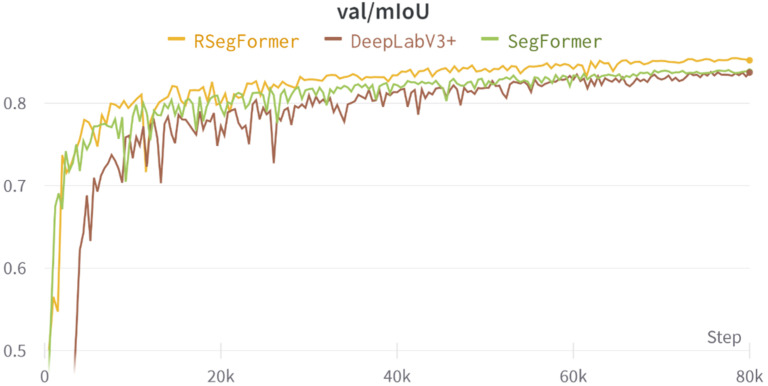
MIoU validation curves for different models.

In this experiment, we split the 1350 disease images obtained using two RLDCP data augmentations into a training and validation set in a ratio of 8:2. Figure 6 shows the MIoU validation curves for the Deeplabv3+, Segformer and RSegformer models. It can be seen that compared with the Deeplabv3+ and Segformer models, the RSegformer model starts with relatively high accuracy, converges faster, has less oscillation and has the highest MIoU throughout, indicating that the network model has high stability and generalisability.

Table 4 shows the number of parameters, flops and their comparative performance in rice leaf disease segmentation for the three models. The RSegformer was experimentally shown to outperform DeepLabv3+ and Segformer in terms of MIoU. Relative to the Segformer model, RSegformer improved IoU for bacterial blight, blast, brown spot and leaf segmentation by 1.44%, 2.28%, 1.96% and 1.34%, respectively.

To compare the segmentation performance of different models, we calculated the number of parameters and FLOPs of RSegformer and models of different sizes such as DeepLabv3+ (ResNet18), Segformer (MiT-B1) and Segformer (MiT-B2) and obtained MIoU based on the training and validation results. From Table 4, we can see that the RSegformer model has the second highest number of parameters and GFLOPs after Segformer (MiT-B1) but achieves the highest MIoU and achieves a better balance of model accuracy and speed.

**Figure 7:**
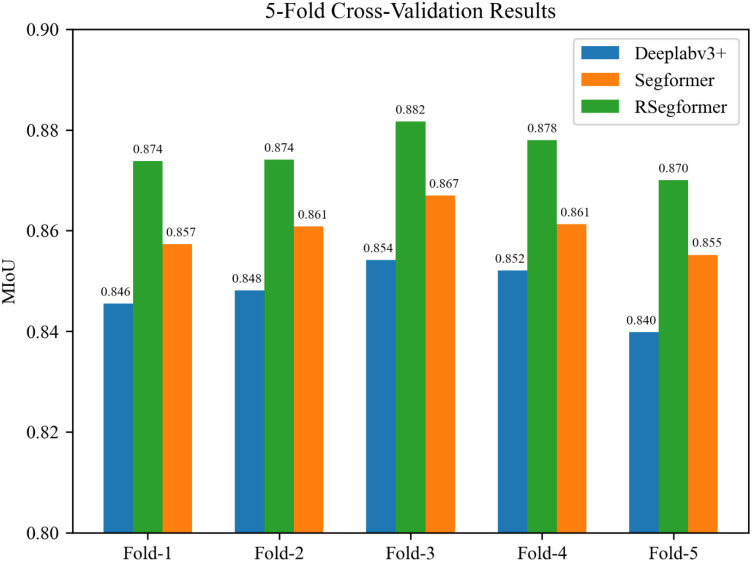
MIoUs for the three models were validated using 5-fold cross-validation. The dataset was divided into 5 parts, set as data-1 to data-5. fold-i is equal to the experimental results obtained by treating data-i as the validation set and the rest of the data as the training set.

In this experiment, a fivefold cross-validation approach was chosen in order to obtain more accurate training results to verify that our chosen model was valid and reliable. Firstly, the dataset obtained after twice using RLDCP data augmentation was divided into five subsets, and in each subset, the number of each level of each disease was equally divided. Secondly, during the training process, one of these five subsets was sequentially used as the validation set and the remaining four subsets were used as the training set for the experiment, constituting five sets of training and validation data. Finally, these five sets of data were trained on each of the three models. It is noticeable that we set the batch size to 4 this time and the total number of iterations trained remained the same. The results are shown in Figure 7.

Firstly, the MIoUs of the three models fluctuated less among the five sets of experiments. Secondly, the differences between the three models were relatively stable in each experiment. Finally, it is evident that RSegformer performed best among the three models, with MIoUs on average 1.5% and 2.5% higher than Segformer and Deeplabv3+, respectively.

To verify that the cross-validation results of the three models were statistically significantly different, and because the crossover experiments for the three models were independent of each other and their results were consistent with continuity, normality and homogeneity of variance, we chose a one-way analysis of variance (ANOVA) and used SPSS software for statistical analysis. The ANOVA results showed that the different models had significantly different effects on MIoU, F = 39.853, *p* = 0.000005, as shown in Table 5. The multiple mean comparison results showed that the RSegformer model was significantly better than Segformer and Deeplabv3+.

### 4.3. Model Ablation Study

The ablation experiments were designed to investigate the effectiveness of the sampling operators on the shunted transformer, CA attention and CARAFE. We compared the IoUs of background, leaf, bacterial blight, blast, brown spot and the overall MIoU, as shown in Table 6. The segmentation performance of the models that did not use these methods was below the maximum accuracy of RSegformer.

Model 1 is the original Segformer model, Model 2 is the model after replacing the encoding part of the Segformer model with shunted transformer and Model 3 is the model after adding CA attention to the middle part of encoding and decoding on top of Model 2. Model 4 is the model after replacing the bilinear up-sampling with CARAFE in the decoding part on top of Model 2.

After replacing the coding backbone with shunted transformer, the segmentation accuracy improved for almost all diseases and leaves and backgrounds except for rice bacterial blight. This may be because SSA has better feature extraction for small targets through multiscale token aggregation, which unifies multiscale feature extraction within a single self-attentive layer and therefore has better segmentation capability for dense micro-miniature disease spots such as brown spots and rice blast.

The segmentation accuracy of rice bacterial blight disease improved with the addition of CA attention. This may be because CA attention takes into account the relationships of the location information in the feature space, which enables the model to capture the long-distance dependence between spatial locations. Therefore, there is an improvement in segmenting images of rice bacterial blight, which has an onset colour similar to that of rice ears and a wide distribution area.

All disease segmentation accuracies improved significantly with the addition of the CARAFE operator. This may be because the CARAFE up-sampling method has a larger perceptual field and can make better use of the surrounding information and also the up-sampling kernel in CARAFE is related to the semantic information of the feature map, enabling up-sampling based on the input content. Thus, it can significantly improve the overall segmentation performance of the network.

When CA attention and CARAFE cooperate, the segmentation accuracy of all diseases except brown spot improved. We determined that this may be because since CARAFE can better capture semantic information, CA attention can better capture feature spatial location information, and the combination of the two to complement each other effectively improves the model’s segmentation performance.

### 4.4. Comparison of Model Inference Results

**Figure 8:**
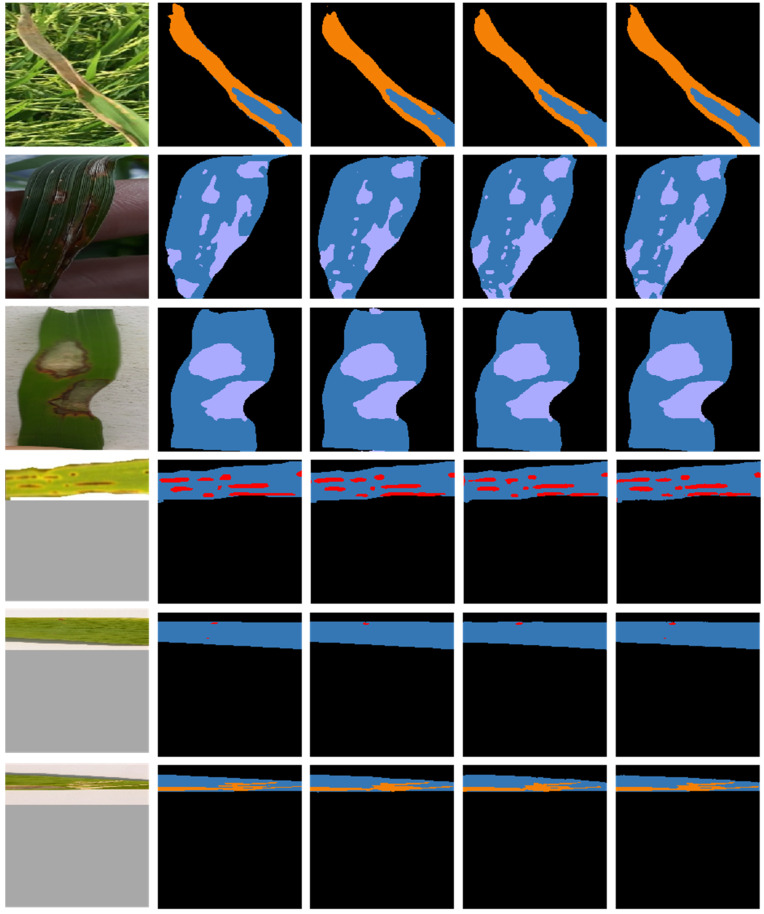
Example inference results for the validation sets on the three models. The first column represents the real images, the second column represents the real labels, the third column shows the inference results for the DeepLabv3+ network model, the fourth column shows the inference results for the Segformer network model, and the fifth column shows the inference results for the RSegformer network model.

To investigate the segmentation performance of Deeplabv3+, Segformer and RSegformer, we analysed the inference results. This is shown in Figure 8. From the first row, it can be seen that RSegformer identifies leaf edge contours better than the other models in the presence of complex background interference. The second row shows that RSegformer can still detect rice blast onset areas and achieve fine segmentation under dark light conditions. In the third row, Deeplabv3+ misdetects the white-grey area above the leaf as a bacterial blight lesion in the segmentation, but Segformer and RSegformer do not, as the rice blast onset area exhibits very similar colour symptoms to the background. In the fourth row, it can be seen that the RSegformer model segmented the blurred edges of brown spot disease very well, in line with the conclusion obtained in the ablation study that CA attention significantly improved the fine segmentation of margins. In the fifth row, we find that Deeplabv3+, and Segformer both show missed detection of fine disease spots. RSegformer can segment accurately, which has important implications for the timely monitoring and early warning of rice diseases. The sixth row uses the synthetic leaf of rice bacterial blight disease and its inference results after data enhancement, and the segmented area of RSegformer was closer to the labelled image.

### 4.5. Comparison of Rice Disease Severity Estimates

**Figure 9:**
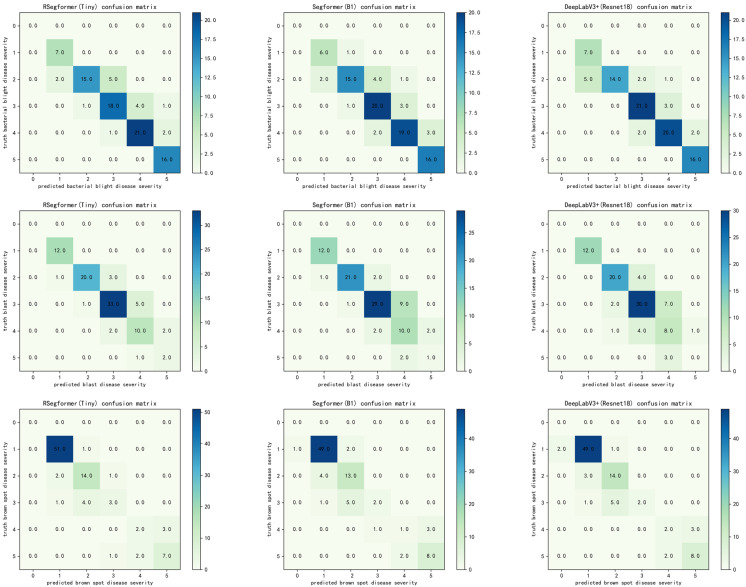
Confusion matrices for the severity classes of different rice diseases under different network models. The first row is the confusion matrix of the three models for the estimation of the severity of rice bacterial blight disease, the second row is the confusion matrix of the three models for the estimation of the severity of rice blast disease and the third row is the confusion matrix of the three models for the estimation of the severity of brown spot disease.

Based on the model segmentation results, rice disease areas and leaf areas can be extracted, the disease percentage of leaf area can be calculated based on the area pixel area and severity classes can be calculated based on the rice disease classification criteria in Section 2.1.4. In this experiment, the confusion matrices of DeepLabv3+, Segformer and RSegformer for determining the severity of rice bacterial blight, rice blast and brown spot, respectively, were compared, as shown in Figure 9. In the confusion matrix, each row represents the correct category, and each column represents the predicted category. RSegformer performs better in rice disease severity estimation.

To improve our understanding of the causes of this phenomenon, we further analysed the misclassification problem. Analysis of the confusion matrix showed that our model was more accurate than other models in grading the severity estimates for the two diseases other than rice bacterial blight. For rice bacterial blight, rice blight and brown spot, 16, 15 and 15 samples were misclassified by RSegformer, respectively, with disease severity overestimated in 12, 10 and 5 of these samples. Possible reasons for this misclassification were blurred leaf edges and similarity of leaf colour to the background, resulting in the segmented leaf area being smaller than the actual area.

RSegformer was below average for accuracy at level 3, with rice bacterial blight and rice blast mostly overestimated at level 4, which we determined was a result of the difficulty in defining the edges of the yellow halo for some diseases, resulting in the model predicting a larger lesion area than the marked area. Brown spot was mostly underestimated at level 2, probably due to the clustering of spots, which made it difficult to split the predicted spots.

## 5. Conclusions

In this study, a semantic segmentation method based on Segformer, shunted transformer encoder, CA attention mechanism and CARAFE up-sampling operator was proposed to identify and segment rice bacterial blight, rice blast and brown spot; improve segmentation accuracy using the RLDCP data enhancement method and calculate the area and number of spots based on the segmentation results; then, the disease severity classification criteria were used to determine the rating. The results show that: (1) the proposed RLDCP data enhancement method outperforms traditional data enhancement methods in generalisation and significantly improves the detection performance of the semantic segmentation model without additional training costs compared with GAN models. (2) The RSegformer semantic segmentation model achieves MIOU of 85.38%, in contrast with DeepLabV3+ and Segformer, with minor increases in the numbers of parameters and computational effort, exceeding DeepLabV3+ by 1.91% and the Segformer-B1 model by 1.43%. (3) The model has greater accuracy in classifying lesion severity on the newly established severity criteria.

The semantic segmentation model proposed in this study achieves pixel-level classifications of different rice diseases and provides a reference for related plant disease detection studies. In the future, we suggest designing semantic segmentation models with higher accuracy and smaller size, expanding the dataset of different disease types and disease stages of rice, producing more fine-grained semantic segmentation labels and adopting more concise and efficient data enhancement methods to achieve rice disease segmentation and severity ranking.

## Figures and Tables

**Table 1:** Dataset structure.

	Bacterial Blight	Brown Spot	Blast	Total
Dataset 1	48	95	50	193
Dataset 2	40	40	0	80
Dataset 3	62	15	100	177
Total	150	150	150	450

**Table 2:** Selected parameters in different phases of the shunted-tiny model. Head indicates the number of heads in a shunted transformer block, $N_{i}$ indicates the number of shunted transformer blocks in a phase, $C_{i}$ indicates the output dimension.

	Stage 1	Stage 2	Stage 3	Stage 4
Layer Name	Shunted Transformer Block	Shunted Transformer Block	Shunted Transformer Block	Shunted Transformer Block
Shunted-Tiny	$$r_{i}=\{\begin{array}{c} 4,\;\;i<\frac{head}{2}\\ 8,\;\;i\geq \frac{head}{2} \end{array}$$	$$r_{i}=\{\begin{array}{c} 2,\;\;i<\frac{head}{2}\\ 4,\;\;i\geq \frac{head}{2} \end{array}$$	$$r_{i}=\{\begin{array}{c} 1,\;\;i<\frac{head}{2}\\ 2,\;\;i\geq \frac{head}{2} \end{array}$$	r=1
	$C_{1}=64,\;\;head=2,\;\;N_{1}=1$	$C_{2}=128,\;\;head=4,\;\;N_{2}=2$	$C_{1}=256,\;\;head=8,\;\;N_{3}=4$	$C_{4}=512,\;\;head=16,\;\;N_{4}=1$

**Table 3:** Comparison of MIoU of different augmentation methods.

	PSPNet	HRNet	OCRNet
Without augmentation	77.52%	78.36%	79.48%
Rotate + Noise augmentation	76.36%	78.09%	78.73%
RLDCP augmentation (once)	82.05%	83.64%	83.60%
RLDCP augmentation (twice)	82.99%	84.70%	84.52%

**Table 4:** Comparison of Deeplabv3+, Segformer and RSegformer models.

	RSegformer	DeepLabv3+	Segformer-B1	Segformer-B2
MIoU (%)↑	85.38	83.47	83.95	84.93
IoU of Background (%)↑	99.33	99.25	99.21	99.33
IoU of Leaf (%)↑	92.08	90.95	90.74	91.64
IoU of Bacterial blight (%)↑	80.91	79.21	79.47	73.65
IoU of Blast (%)↑	79.96	78.73	77.68	79.65
IoU of Brown spot (%)↑	74.61	69.22	72.65	80.61
Params (M)↓	14.36	12.47	13.74	27.48
Flops (G)↓	26.13	54.31	15.94	62.45

**Table 5:** One-way analysis of variance results for different models.

Model	MIoU (%)	F-Test		Multiple Comparisons
	($\bar{x}\pm s$)	F	P	
RSegformer	87.56 ± 0.45	39.853	0.000005	RSegformer > Segformer
Segformer	86.02 ± 0.46			RSegformer > DeepLabv3+
DeepLabv3+	84.80 ± 0.54			Segformer > DeepLabv3+

**Table 6:** MIoU comparison in ablation study when removing some blocks.

	Model 1	Model 2	Model 3	Model 4	RSegformer
MIoU	83.95%	84.50%	84.43%	85.13%	85.22%
Background	99.21%	99.27%	99.26%	99.31%	99.35%
Leaf	90.74%	91.20%	91.43%	91.61%	92.15%
Bacterial blight	79.47%	79.31%	79.65%	80.16%	80.46%
Blast	77.68%	78.44%	78.23%	79.57%	79.67%
Brown spot	72.65%	74.29%	73.60%	75.03%	74.50%

## Data Availability

The three varieties of diseased rice leaf images and masks used in this study are available at https://www.kaggle.com/datasets/slygirl/rice-leaf-disease-with-segmentation-labels (accessed on 3 November 2022) and can be shared on request.
